# Magnitude and risks of overweight/obesity among adults in Welkite town, Southern Ethiopia: A community based cross-sectional study

**DOI:** 10.1371/journal.pone.0275014

**Published:** 2022-09-21

**Authors:** Alemayehu Fikre, Muze Shehmolo, Negussie Boti, Bilcha Oumer, Belaynesh Tenalem, Sahle Kibru, Gebremaryam Temesgen, Zeleke Gebru

**Affiliations:** 1 Gurage Zone Health Department, Welkite, Gurage Zone, Southern Ethiopia, Ethiopia; 2 Kibet Primary Hospital, Worabe, Silte Zone, Southern Ethiopia, Ethiopia; 3 School of Public Health, College of Medicine & Health Sciences, Arba Minch University, Arba Minch, Ethiopia; 4 School of Public Health, College of Medicine and Health Sciences, Wolaita Sodo University, Wolaita Sodo, Ethiopia; 5 Department of Midwifery, College of Health Sciences, Arba Minch, Ethiopia; 6 Welkite Health Center, Welkite, Gurage Zone, Southern Ethiopia, Ethiopia; 7 Department of Midwifery, College of Medicine and Health Sciences, Arba Minch University, Arba Minch, Ethiopia; Università degli Studi di Milano, ITALY

## Abstract

**Background:**

Currently, adult overweight/obesity affects a high proportion of the population in low and middle-income countries, mostly in urban areas. Although some studies have been conducted on overweight/obesity in Ethiopia, most of them have focused on school children and adolescents, and there is limited evidence of overweight/obesity among adults at the community level. Therefore, the present study aimed to assess the magnitude of overweight/obesity and risk factors among adults in Welkite town, Southern Ethiopia.

**Methods:**

A Community-based cross-sectional study was done among 524 adults aged 18 and more years in Welkite town, Southern Ethiopia, from February through March 2020. A multistage sampling technique was undertaken to recruit study participants. An interviewer-guided structured questionnaire was used for data collection. Overweight or obesity was identified using body mass index. The bivariate and multivariate analyses were employed to see an association using binary logistic regression.

**Results:**

The magnitude of overweight and obesity was 22.2% (95% CI: 0.19, 0.26). Being female (AOR = 2.40, 95% CI: 1.34, 4.27), age group 30–47 years (AOR = 3.26, 95% CI: 1.52, 6.97) and 48–66 years (AOR = 2.56, 95% CI: 1.07, 6.08), average monthly income (AOR = 2.64, 95% CI: 1.51, 4.60), had own transport (AOR = 2.48, 95% CI: 1.03, 5.93), eating meat ≥ four times per week (AOR = 3.33, 95% CI: 1.03, 10.74), not involve vigorous-intensity activity (AOR = 2.96, 95% CI: 1.55, 5.64), spent sitting or reclining ≥181 minutes per day (AOR = 1.88, 95% CI: 1.08, 3.26), and consuming alcohol (AOR = 2.23, 95% CI: 1.29, 3.82) were risks for overweight and obesity.

**Conclusions:**

The magnitude of overweight and obesity among adults was high. Factors such as being female, increasing age, physical inactivity, having own transportation, high average monthly income, eating meat, sitting or reclining more and equal to 181+ minutes per day, and consumption of alcohol increased the risk of overweight and obesity significantly. Hence, preventive interventions focusing on females, age groups of 30-66yrs, encouraging Physical activity, reducing meat frequency, and reducing alcohol consumption are essential to prevent the emergence of adulthood overweight/obesity.

## Introduction

Obesity and overweight are defined as fat accumulation in the body abnormally or excessively that may affect health [1]. Overweight and obesity are classified as a body mass index (BMI) from 25 to 29.9 kilograms (kg) per meter squared (m2) for overweight and ≥30 kg/m2 for obesity [2, 3]. BMI is a cheap, suitable, and reliable measure used to estimate obesity and overweight at the population‐level, and is calculated by dividing body weight in kg by ht in meter square [4].

Obesity is increasing rapidly across the globe, and it also affects a high proportion of the population of low-income countries [5]. Worldwide, about 2 billion individuals (age ≥18 years) were overweight; of these more than 600 million were obese in 2014. The occurrence of obesity is rising worldwide [1]. Being overweight and obesity were previously known as the problems of the industrialized world, despite the problems increasing dramatically in low- and middle-income countries, mostly in urban areas [4].

Rates of overweight and obesity increase in Sub-Saharan Africa (SSA) and rates of non-communicable diseases (NCDs) are on the rise as well. Obesity and overweight occur when people consume more calories than they burn through time [1, 6]. The rapid nutrition transition can cause the rise of obesity and related diseases in less developed countries [7]. Diets with a high density of energy, rich in fats and sugars, refined carbohydrates, and low fibers have a greater possibility of unhealthy weight gain [6, 8]. The food supply of each country has been influenced by several factors in the past 10 to 15 years. The food system across developing nations in most urban and an increasing proportion of rural areas has changed radically with the globalized distribution of technology processed food, transportation and marketing, mass media, and the flow of capital and services. These complex changes are reflected in the occurrence of obesity besides malnutrition even in the same households (HHS) [8]. They are also the result of poor physical activity due to the increasing sedentary occupation, transition to vehicle-based transportation, increasing urbanization, and development of the economy [1, 7].

Obesity and overweight are mainly exposed to non-communicable diseases(NCDs), such as type II diabetes, heart disease, hypertension and stroke, and cancer [9]. Based to the World Health Organization (WHO), in the decade magnitude of global NCDs will increase by 17%, and in the African region by 27%. They are now causing about 60% of the deaths together with injuries and violence globally. Except in Sub-Saharan Africa, NCDs are the front cause of death, and the majority of them occur in economically developing countries [10]. Premature death from NCDs is higher in low-income countries than in high-income countries [11].

Many studies have shown the number of overweight and obesity is ongoing of increasing in both developed and developing countries [4, 5, 12]. However, overnutrition and undernutrition are remaining double the burden of developing countries [13]. In addition, NCDs and communicable diseases are the dual burden of developing countries and countries experiencing socioeconomic and nutritional transition, predominantly in the urban population [14–16].

Globally, more than 1.9 billion adults were overweight and obese in 2014 [1]. Between the years 1980–2013, rates of overweight and obesity among adults have increased for men and women from 29 to 37 and from 30 to 38 percent respectively. The world’s highest proportion of obese people (13%) lives in the United States and 15% in both China and India. By 2013, the top rates (58% of men and 65% of women aged≥20) of overweight and obesity has reported in the Middle East and Northern Africa. In SSA, the highest rate (42%) of obese women was found in South Africa [17].

According to the Ethiopian demographic and health survey (EDHS), the rate of overweight/obesity increased from 3% in 2000 to 8% in 2016 among women and from 2.5% in 2011 to 3.5% in 2016 among men at the national level. The survey also indicated that overweight/obesity in Addis Ababa has increased from 19.9% in 2011 to 29.4% in 2016 among women and from 12.4% to 19.6% among men in the same year [18, 19]. Most of the studies showed the proportion of overweight and obesity higher in women than men [20–23], and urban settings have a higher percentage of overweight and obesity than rural settings [24–27]. The result of different studies depicts that overweight and obesity had a direct relationship with age (the proportion of BMI increases with increased age) [28–30].

Being overweight and obese leads to serious health problems. Elevated BMI increases the risk factor for chronic diseases such as cardiovascular disease, diabetes, musculoskeletal disorders–especially osteoarthritis, and some malignancy (uterine, breast, and colon). Obesity increases premature death in children and disability in adulthood [31, 32]. The percentage of NCDs has increased from 20% in 2000 to 31% in 2015 (the change is 56%) in low-income countries [33]. The fifth leading risks for world mortality are attributable to Overweight and obesity. Yearly about 3 million adults die because of being overweight or obese. Besides, the burden of 44% of diabetes, 23% of ischemic heart disease, and between 7% and 41% of certain cancer are attributable to overweight and obesity [6]. NCDs are estimated to account for 30% of total deaths in Ethiopia [34]. According to the health management information system report of the Gurage zone, chronic diseases such as hypertension account for 3.4% of all morbidity in 2010 EFY and 4.3% in 2011 EFY, and diabetes accounts for 1.2% in 2010 Ethiopian fiscal year(EFY) and 1.4% in 2011 EFY. This report in Welkite town (2011 EFY) shows 13 persons per 1000 population had hypertension and related diseases and 6 persons per 1000 population had diabetes mellitus.

Although very few studies have been conducted on overweight/obesity in Ethiopia, most of them have focused on school children and adolescents [35–37], and there is limited evidence on overweight/obesity among adults, under 5, pregnant women, & lactating women at the community level (19). And this study is the first at the community level among adults of urban residents in Ethiopia. Results might contribute to the development of appropriate planning & making informed decisions by those concerned institutions & organizations in promoting healthy body weight. The result and recommendations of the study can also help policymakers and programmers to know the common and avoidable factors and design a feasible and convenient programmatic approach for adults in improving their body weight. Finally, this study will help as a reference for other studies and may trigger other researchers to conduct a similar study in addressing wider areas of the country. Therefore, this study aimed to assess the magnitude of overweight/obesity and risk factors among adults in Welkite town, Southern Ethiopia.

## Materials and methods

### Study area, design, and period

A community-based cross-sectional study design was conducted in Welkite town from 03-Feb through 01-Mar-2020. Welkite is one of the two towns administrative in the Gurage zone which is found 259 km in the North East of Hawassa the capital city of Southern nations, nationalities, and People Region. The town is located 155 km away from Addis Ababa the capital city of Ethiopia. The town has 5 administrative kebeles. There are two government health centers and 10 private clinics. Based on records from the Central Statistics Agency of Ethiopia 2007, its population in 2011 EFY is 43,445, of which 21,070 are male [38]. The town is found in the altitude range from 1910 to 1935 m above sea level, and it covers an area of 7260 ha. The range of annual minimum and maximum temperature of the district is 25 and 29°C, respectively. The range of annual rainfall is between 800–1000 mm. The area is found at a latitude and longitude of 08°17’00’N 37°47′00’ E respectively. According to the Gurage Zone Agriculture and Rural Development Department, the common crops grown around Welkite town are teff, maize, red pepper, sorghum, khat, and ‘enset’.

### Source and study population

All adult populations aged 18 years and above living in the Welkite town were the source populations and all adults living in randomly selected HHs in Welkite town were the study populations.

### Inclusion and exclusion criteria

All adults aged 18 years and above who have lived in the town for six months or longer were included in this study and those with serious illness, mental illness, unable and/or unwilling to respond to the questionnaire, and women with known pregnancy were excluded.

### Sample size determination

Sample size determination for the prevalence of overweight and obesity among adults in Welkite town was calculated using the single population proportion formula. The proportion of obesity of 19.2% was taken from the study conducted among adults in Kinondoni municipal district, Dar Salaam Tanzania in 2011 [22]:

$$\text{n}={\left({\text{Z}}_{1-\alpha /2}\right)}^{2}*\text{p}*\left(1-\text{p}\right)/{\text{d}}^{2}={\left(1.96\right)}^{2}\left(0.192\right)\;\left(0.808\right)/{\left(0.05\right)}^{2}=238$$

Where:—n = sample size. d = Margin of error tolerated = 0.05 was taken for this study.
P = Anticipated population proportion of the obesity (19.2%) ^22^
Z_1- α/2_ = standard normal distribution at 95% Confidence Interval (CI) = 1.96. A design effect of two ^22^ and–a non-response rate of 10% was considered. Thus, n = (2*238) + (0.1*2*238) = 524.

Sample size determination for factors associated with overweight and obesity among adults in Welkite town was determined using the study conducted in Dar es Salaam-Tanzania [22], and variables such as sex, socioeconomic status, and physical activity were taken for the calculation. (Epi info7 was used for calculation) (Table 1).

**Table 1:** Sample size determination for the second objective in Welkite town, Southern Ethiopia, 2020.

Variables		Confidence level	Power	Percent in-unexposed	Adjusted OR	Non-response rate (10%)	The sample size for both groups
Sex	Male	95%	80%	9	3.6		191
	Female					17	
Socioeconomic status	Low	95%	80%	11.3	2.6		308
	High					28	
Physical activity	Light	95%	80%	26	0.4		317
	Vigorous					29	

By taking the maximum sample size (317) from Table 1 and adding 10% non-response, the total n = 317 + (0.1*317) = 349. To achieve overall stated objectives, the larger sample size estimated by prevalence and factors was taken i.e., 524.

### Sampling procedures/techniques

A multistage sampling technique was employed to select the study households (HHS). First, Welkite town was selected with the Simple Random Sampling (SRS) technique between two town administrations. Second, out of five kebeles, two kebeles were selected with the SRS technique. The numbers of the sample size required from the selected kebeles were determined by using the proportionate sample size method. The total number of HHs (N) in the selected two kebeles was about 2312. Finally, the study HHs were selected by systematic random sampling method at regular intervals. We determined the sampling interval (K) by using the formula K = N/n, and we found 2312/524 = 4.4. Therefore, the investigator stood at the center of the village and determined the starting direction with the SRS technique. Using a lottery method one of the first HHs within the first interval was selected and every 4th HHs were included in the study until the required sample size was attained.

### Definitions and measurement

According to Communicable Disease Control categorical classification, the study participants were classified based on their BMI as follows [39]

**Overweight**: BMI = 25–29.9 kg/m2

**Obese**: BMI ≥30 kg/m2

**Typical week** means a week when a person is doing vigorous or moderate-intensity activities and not an average over a period; valid responses range from 1–7 days [40].

**Vigorous-intensity activities** are activities that require hard physical effort and cause large increases in breathing or heart rate. Doing such activities or sports for at least 10 minutes continuously, for example, carrying or lifting a heavy load, digging or construction work, running or jogging and football playing [39].

**Moderate-intensity activities** are activities that require moderate physical effort and cause small increases in breathing or heart rate, for example, undertaking volleyball, brisk walking or hiking, carrying or lifting light loads, swimming, and cycling, for at least 10 minutes continuously [39]. **Intensity** is how hard a person works to do the activity. The intensities most often examined are moderate-intensity and vigorous-intensity [41].

**Sedentary behavior** is considered when an individual spends sitting or reclining on a typical day for more than 181 minutes [42].

### Data collection tools and procedures

The data were collected using an interviewer-guided structured questionnaire. Most of the questionnaire was adapted from the WHO global physical activity questionnaire and nutritional status and lifestyle questionnaire from RenaiSante Institute of Integrated Medicine [39, 43] and the rest was partially adapted from the literature such as Kinondoni municipal district, Dar es Salaam Tanzania [22] and Mayo area in Khartoum State-Sudan [20]. Frequency of meat, cheese, vegetables, fruits, alcohol, bread, and cereals, physical activity intensity, walking or using a bicycle, doing sports, fitness, or recreational activities continuously, and time usually spend sitting or reclining on a typical day were taken from the reference mentioned.

The weight and height of the respondents were measured from a house-to-house investigation. Weight was measured with a precision of 0.1 kg with an adult digital portable measuring scale, and height was measured with a precision of 0.5 cm with a portable height measuring rod in a standing position after participants wearing light clothes and without shoes. The BMI of respondents was calculated by dividing weight in kg by height in m2.

### Data quality assurance procedures

The questionnaire was first developed in English and translated into the local language (Amharic) and it was checked by different experts for consistency of translation of the language. Three days of training were given to four data collectors and one supervisor on data collection and anthropometric measurement by the principal investigator and other health professionals. All trainees were health professionals and at least diploma holders to ensure the quality of data. The questionnaire (5%) was pre-tested for appropriateness before major data collection in another kebele which was not included in the study. As a first-line quality control check, we focused on completing questionnaires. Once checked for completeness and accuracy, data entry clerks typed and entered data into EPI-Info 7.0 using a given identity number. Two independent data clerks performed a double-entry of 3% of the questionnaire to check for consistency.

### Data analysis procedures

Frequency, percentages, and proportions were cross-tabulated by using variables included in our study. Data were checked, coded, and entered into a predetermined template using Epi-info version 7 and exported to and analyzed using IBM SPSS Statistics for Windows, version 21 (IBM Corp., Armonk, N.Y., USA). A bivariate analysis was used to see the association between each independent and dependent variable by estimating the crude odds ratio along with 95% CI. Multivariate analysis was applied following bivariate analysis with a p-value of < 0.25 ^22^ to adjust for the effect of confounders using multiple binary logistic regressions, both crude odds ratio (COR) and adjusted odds ratio (AOR) with 95% CI was reported. Finally, statistical significance was reported at a p-value ≤ 0.05 with 95% CI. Multicollinearity was checked and Hosmer and Lemeshow’s test goodness of model fit was 0.879.

### Ethical consideration

Before actual activities, ethical clearance was obtained from the Institutional Research Ethics Review Board (IRB) of Arba Minch University with Ref. no: IRB/046/10. Official letters of permission were obtained from the Gurage zone health department and Welkite health office. This study was conducted following the Declaration of Helsinki. After clearly explaining the purpose of the study, the harm and the benefit, the confidentiality, and the rights of the study subject, written informed consent was obtained from each study participant. To maintain confidentiality, no personal identifiers were used on data collection forms and the recorded data were not accessed by a third person, except the principal investigators.

## Results

### Socio-demographic characteristics of participants

A total of 513 adults (aged 18 and above) participated in the study with a response rate of 97.9%. The mean age of the respondents was 37.6 years (37.6 ± 14.2SD). The majority 218 (42.5%) of the respondents were in the age group between 30 and 47. Among the participants males were 271 (52.8%), Orthodox religion followers 220 (42.9%), civil servants 149 (29%), Gurage ethnicity 202 (39.4%), married 356 (69.4%) and 170 (33.1%) attended College or University education. About 277 (54%) of the respondents had greater than or equal to five people dwelling in their houses. Only 33 (6.4%) owned personal automobiles. The mean monthly average family income was 3200 Ethiopian Birrs. (Table 2).

**Table 2:** Sociodemographic characteristics of the study participants (n = 513).

Variable	Numbers	Percentage
Sex		
Male	271	52.8
Female	242	47.2
Age (in years)		
< 30	159	31.0
30–47	218	42.5
48–66	110	21.4
67+	26	5.1
Religion		
Orthodox Christian	220	43.0
Muslim	152	29.6
Protestant	76	14.8
Adventist	53	10.3
Catholic	12	2.3
Occupation		
Civil servant	149	29.0
Housewife	79	15.4
Trader	66	12.9
Private employee	59	11.5
Student	57	11.1
Daily laborer	55	10.7
Farmer	26	5.1
Jobless	22	4.3
Ethnicity		
Gurage	202	39.4
Amhara	182	35.5
Kebena	58	11.3
Oromo	48	9.3
Other	23	4.5
Marital status		
Single	111	21.6
Married	356	69.4
Widowed	32	6.2
Divorced	14	2.8
Educational level		
No formal education	64	12.5
Primary (Grade 1–8)	131	25.5
Secondary (Grade 9–12)	148	28.8
College or University	170	33.1
Do you have your automobile?		
Yes	33	6.4
No	480	93.6
Average monthly income		
Below the mean(<3200Birrs)	318	62
Greater or equal to the mean (3200+Birrs)	195	38
Family size		
< 5	236	46
5+	277	54

### Eating habit

Regarding eating habits, about half 273(53.2%) ate meat once a week, 269(52.4%) ate vegetables 2–3 times a week, 226 (44.1%) drink milk, cheese, and/or yogurt once a week, and 275 (53.6%) ate sugar and sweets 6 and more times a week. A greater proportion of 505 (98.4%) respondents had meals greater or equal to three times a day (Table 3).

**Table 3:** Eating habits of respondents in Welkite town of Southern Ethiopia, 2020, [n = 513].

Variable	Numbers	Percentage
How often did you eat meat or eggs in a week?		
Did not eat in a week	123	24.0
Once a week	273	53.2
Two-three times a week	93	18.1
Four and more times a week	24	4.7
How often did you eat Milk, Cheese, and Yogurt in a week?		
Did not eat in a week	219	42.7
Once a week	226	43.9
Two-three times a week	53	10.5
Four and more times a week	15	2.9
How often did you eat Sugar and sweets in a week?		
Did not eat in a week	40	7.8
Once a week	28	5.5
Two-three times a week	77	15.0
Four-five times a week	93	18.1
Six and above times a week	275	53.6

### Physical activity and sedentary behavior

Out of 513 participants, 186(36.3%) did vigorous-intensity work at least 10 minutes a day, 455 (88.7%) walked or used a bicycle to travel to and from places for at least 10 minutes continuously, and about 201 (39.2%) experienced sedentary behavior like sitting at a desk, sitting with friends, using a car to move place to place, reading, playing cards, or watching television (Table 4).

**Table 4:** Physical activities and sedentary behavior of the study participants (n = 513).

Variable	Numbers	Percentage
Vigorous-intensity work for at least 10 minutes continuously?		
Yes	186	36.3
No	327	63.7
Moderate-intensity works that for at least 10 minutes continuously?		
Yes	146	28.5
No	367	71.5
Did you walk or use a bicycle for at least 10 minutes continuously to get to and from places?		
Yes	455	88.7
No	58	11.3
Did you do any vigorous-intensity sports, fitness, or recreational activities for at least 10 minutes continuously?		
Yes	27	5.3
No	486	94.7
Moderate-intensity sports, fitness, or recreational (leisure) activities that for at least 10 minutes continuously?		
Yes	135	26.3
No	378	73.7
How much time did you usually spend sitting or reclining on a typical day?		
≤ 180 minutes	312	60.8
181+ minutes	201	39.2

### Alcohol use and smoking cigarettes

According to the study, 147 (28.7%) of the respondents consumed alcohol, and 23 (4.5%) of respondents smoked a cigarette (Table 5).

**Table 5:** Alcohol use, smoking cigarettes, and chronic conditions of respondents in Welkite town of Southern Ethiopia, 2020, [n = 513].

Variable	Numbers	Percentage
Have you ever drunk alcohol?		
Yes	147	28.7
No	366	71.3
Have you ever smoked a cigarette?		
Yes	23	4.5
No	490	95.5

### Prevalence of overweight/obesity

The overall prevalence of overweight was 18.1% (95% CI: 0.15, 0.22) and obesity was 4.1% (95% CI: 0.02, 0.06). The combined prevalence of overweight and obesity was 22.2% (95% CI: 0.19, 0.26). The highest prevalence of overweight or obesity was seen in adults aged 30–47 years, followed by adults aged 48–66 years. The prevalence of overweight in males was 7.8% and in females was 10.3%, obesity in males was 1.2% and in females was 2.9%, and the combined prevalence of overweight and obesity in males was 9% and in females was 13.2%.

### Factors associated with overweight/obesity

In the bivariate analysis, being female; adults aged 30–47 years; being civil servants, housewives, and traders; being married and widowed; average monthly income was greater or equal to the mean (3200 birrs), and having their transport were factors crudely associated with obesity or overweight.

In the multivariate analysis, the females were 2.4 times more likely to be overweight or obese than males; people in age groups of 30–47, and age groups of 48–66 years were more likely to be overweight or obese compared to the age group of less than 30 years; the respondents who earned average monthly income greater or equal to the mean (3200 birrs) were at higher risk to be overweight or obese; the respondents who had own transportation was 2.5 times more likely to be overweight or obese compared to those who did not have own transportation; the participants who ate meat or eggs four and above times a week were 3.3 times more likely to be overweight or obese than those who never ate; those were not involved in vigorous-intensity work 3 times more likely to be overweight or obese than active workers; the odds of overweight or obesity for people spending by sitting or reclining greater or equal to 181 minutes per day are 88% more than in those physically active people; and the odds of overweight or obesity among alcohol consumers was 2.2 times more than respondents who did not take alcohol (Table 6).

**Table 6:** Factors associated with overweight or obesity among adults in Welkite town, Southern Ethiopia, 2020, [n = 513].

Variables	Overweight/Obesity		COR (95% CI)	AOR (95% CI)
	Yes	No		
	n (%)	n (%)		
Sex				
Male	46 (9%)	225(43.9%)	1	1
Female	68(13.3%)	174(33.9%)	1.91(1.25, 2.91) *	2.40(1.34, 4.27) **
Age				
< 30	15(2.9%)	144(28.1%)	1	1
30–47	61(11.9%)	157(30.6%)	3.73(2.03, 6.85) *	3.24(1.51, 6.92) **
48–66	32(6.2%)	78(15.2%)	3.94(2.01, 7.71) *	2.56(1.07, 6.09) **
67+	6(1.2%)	20(3.9%)	2.88(1.00, 8.27) *	1.62(0.42, 6.09)
Marital status				
Single	10(1.9%)	101(19.7%)	1	1
Married	93(18.1%)	263(51.3%)	3.57(1.78, 7.13) *	1.30(0.49, 3.39)
Widowed	8(1.6%)	24(4.7%)	3.37(1.20, 9.43) *	1.38(0.35, 5.43)
Family monthly income				
< 3200 birrs	47(9.2%)	271(52.8%)	1	1
3200+ birr	67(13.1%)	128(25%)	3.02(1.96, 4.63) *	2.64(1.51, 4.60) **
Having own personal automobile				
No	97(18.9%)	383(74.7%)	1	1
Yes	17(3.3%)	16(3.1%)	4.20(2.04, 8.60) *	2.48(1.03, 5.93) **
Eating meat or eggs per week?				
Did not eat	22(4.3%)	101(19.7%)	1	1
Once a week	61(11.9%)	214(41.7%)	1.31(0.76, 2.25)	1.13(0.56, 2.26)
2–3 times	21(4.1%)	72(14%)	1.34(0.68, 2.61)	0.60(0.26, 1.37)
4 and above	10(1.9%)	12(2.3%)	3.83(1.46, 9.96) *	3.33(1.03, 10.74) **
Involved vigorous-intensity activity/work				
Yes	19(3.7%)	167(32.6%)	1	1
No	95(18.5%)	232(45.2%)	3.60(2.11, 6.12) *	2.96(1.55, 5.64) **
Walked or used a bicycle				
Yes	91(17.7%)	364(71.0%)	1	1
No	23(4.5%)	35(6.8%)	2.63(1.48, 4.66) *	1.65(0.79, 3.39)
Time usually spent sitting or reclining on a typical day				
≤ 180 minutes	55(10.7%)	257(50.1%)	1	1
181+ minutes	59(11.5%)	142(27.7%)	1.94(1.275–2.957) *	1.88(1.086–3.263) **
Have you ever drunk alcohol?				
No	68(13.3%)	298(58.1%)	1	1
Yes	46(9%)	101(19.7%)	2.0(1.289–3.090) *	2.23(1.296–3.828) **

**NB.** *P<0.25,

**P<0.05;

AOR: adjusted odds ratio; COR: crudes odds ratio; 1 reference category, CI confidence interval.

## Discussion

The magnitude of overweight and obesity in adults aged 18 years and above in Welkite town was 22.2%; overweight 93(18.1%), and obesity 21(4.1%). Sex, age, average monthly family income, having own personal transport, eating meat or eggs, doing vigorous-intensity work, sedentary ways of life, and drinking alcohol were independent factors associated with overweight/obesity.

The overall magnitude of overweight and obesity in adults aged 18 years and above in Welkite town was 22.2% (95% CI: 0.19, 0.26). This was found to be higher than the EDHS report of 2016, but lower than the result of urban areas [18]. But it is in line with a study conducted in the Democratic Republic of Congo, Benin, and Malawi [27, 44, 45]. However, the prevalence is lower than a study in Kenya, Rwanda, Uganda, and the Central Africa Republic based on the study conducted in Sun-Saharan Africa [27].

Most of the studies conducted in different countries showed that a prevalence of a higher BMI is observed among women than men. Based on the results of this study, the prevalence of overweight and obesity among females is higher than in males. This finding is supported by EDHS reports of 2016 [18]. Similarly, the studies in Sudan, Dar as Salaam-Tanzania, United States, and Benin reported a higher proportion of overweight or obesity among females than men [20, 22, 29, 44]. The variation might be explained by different distribution in risk factors (e.g., biological variation-women gain weight during menopause, physical activity, and work intensity) between women and men across the community [46, 47].

This study identified the odds of being overweight or obesity are increased with increasing age. This finding is in line with other similar findings in Ghana [48], Benin [44], Malawi [45], and South Sudan [49]. The increase of overweight or obesity with age might be due to the degree of physical activity decrease, and hormonal changes occur during aging in both sexes. Accordingly, decreasing energy expenditure, accumulation of fat, and weight gain might be increased [50].

The finding in the present study showed a statistical association between monthly average income and overweight or obesity. This is in line with other similar findings in Kabul Citizens-Afghanistan, Saudi Arabia, and South Sudan [23, 24, 49]. On the contrary, people with the lowest income and socioeconomic group were more likely to be obese [51, 52]. The difference might be variations in methodology, study time, and geographical location. In addition, participants with the highest income in the study area might be increased food intake of high calories, reduce physical activity and increase sedentary lifestyles [53].

The respondents who had their transportation were more than twice to develop overweight or obesity compared to those who did not own personal transportation. This argument is supported by a cross-sectional study conducted in Bahir Dar city [37]. This might be because the respondents who had their transport may use their vehicle to travel to or from places. As a result, energy expenditure in the body has fallen and body weight gain has increased greatly [5].

The findings of this study depicted that the respondents who ate meat or eggs four and more times per week were more than three times more likely to be overweight or obese. This finding is supported by a study conducted in the United States [54]. The finding might be explained the respondents who consumed meat more frequently are at increased risk of getting obese or overweight because meat contains a high proportion of protein and fat [7].

Physical activity was negatively associated with obesity and overweight in this study. The odds of overweight or obesity among physically inactive people were higher compared to physically active people. This finding is supported by the study among adults in the Mayo area in Khartoum State–Sudan, Afghanistan, Ghana, and Turkey [20, 23, 48, 55]. This might be explained by the fact that physical activities increase people’s total energy expenditure, and it can keep energy balance or weight loss [2, 41].

This study revealed that the respondents who spent their time watching TV or films, playing games, using the computer or internet, sitting at a desk, sitting with friends, traveling in a car, or reading greater or equal to 181 minutes a day were more likely to be overweight or obese. This finding is in line with a study among adults in the Mayo area in Khartoum State-Sudan and a study among women in Addis Ababa [20, 28]. This might be explained that sedentary behavior increases physical inactivity. Consequently, energy balance in the body can be altered; more calories might be deposited, and body weight considerably increased [2, 5].

The present study finding showed that the respondents who took alcohol were more odds of being overweight or obese as compared to those who did not take alcohol. This is supported by a cross-sectional study conducted in Accra Metropolis, Ghana, and Turkey [48, 55]. But, the finding in Dar es Salaam Tanzania revealed no association between alcohol and obesity [22]. This might be explained by the fact that alcohol consumption leads to a positive energy balance because the alcohol they consumed quickly turned to energy; drinking alcohol also seems to trigger impulsive eating behaviors, and alcohol might be decreased fat breakdown and can stimulate its synthesis and deposition [56].

### Strength and limitation of the study

Being a community-based study and involving adults of both sexes can fill the knowledge gap that existed on adults overweight and obesity in the country & in particular in the study area. Nutritional problems are new and emerging challenges to the community, particularly in developing countries, hence these findings address many variables that we can’t find elsewhere in a single study where we effectively control the confounding effects. And also, Strengthen the evidence of how overweight and obesity is increasing in low-income status since >60% of the participants have an average monthly income below the mean (<3200Birrs or 53 dollars per month). And this provides the significance of the problem of populations having different feeding cultures which are mainly based on “Enset” when compared with the other parts of the country.

The cross-sectional study might not be strong enough to determine the direct cause-and-effect relationship between risk factors and outcome. There might be recall bias during data collection by participants, especially the frequency of food they eat, and the time they spent sitting or reclining. There might be also social desirability bias among respondents interviewed, for example, family income, alcohol consumption, or smoking cigarette. Genetic changes, diseases, and drugs that affect body weight gain were not addressed in this study. The proportion taken to calculate the sample size was taken in Tanzania which is considered as having a nearly similar environment. Food quantity was not addressed. And smoking cigarettes and alcohol current status were not assessed.

## Conclusions and recommendations

The magnitude of overweight and obesity among adults was high. Factors such as being female, increasing age, physical inactivity, having own transportation, high average monthly income, eating meat, sitting or reclining more and equal to 181+ minutes per day, and consumption of alcohol increased the risk of overweight and obesity significantly.

Hence, preventive interventions focusing on females, age groups of 30-66yrs, encouraging Physical activity, reducing meat frequency, and reducing alcohol consumption are essential to prevent the emergence of adulthood overweight/obesity.

## Supporting information

S1 Raw data (SAV)
